# Supplementary material for: Abundant mRNA m1A modification in dinoflagellates: a new layer of gene regulation
Source: EMBO Rep. 2024 Sep 2;25(11):5. doi: 10.1038/s44319-024-00234-2 (PMC11549093; doi:10.1038/s44319-024-00234-2)
Supplement: Supplementary file 1 — Appendix [file 44319_2024_234_MOESM1_ESM.pdf]

APPENDIX

Abundant mRNA m^{1}A modification in dinoflagellates: a new layer of gene regulation

Chongping Li[1, #], Ying Li[1,2, #], Jia Guo[3,4, #], Yuci Wang[1, #], Xiaoyan Shi[1], Yangyi Zhang[1], Nan Liang[1], Honghui Ma[5], Jie Yuan[2, *], Jiawei Xu[3,4, *], Hao Chen[1, *]

Table of contents

Appendix Figure S1 Page 2
Appendix Figure S2 Page 3
Appendix Figure S3 Page 4
Appendix Figure S4 Page 5

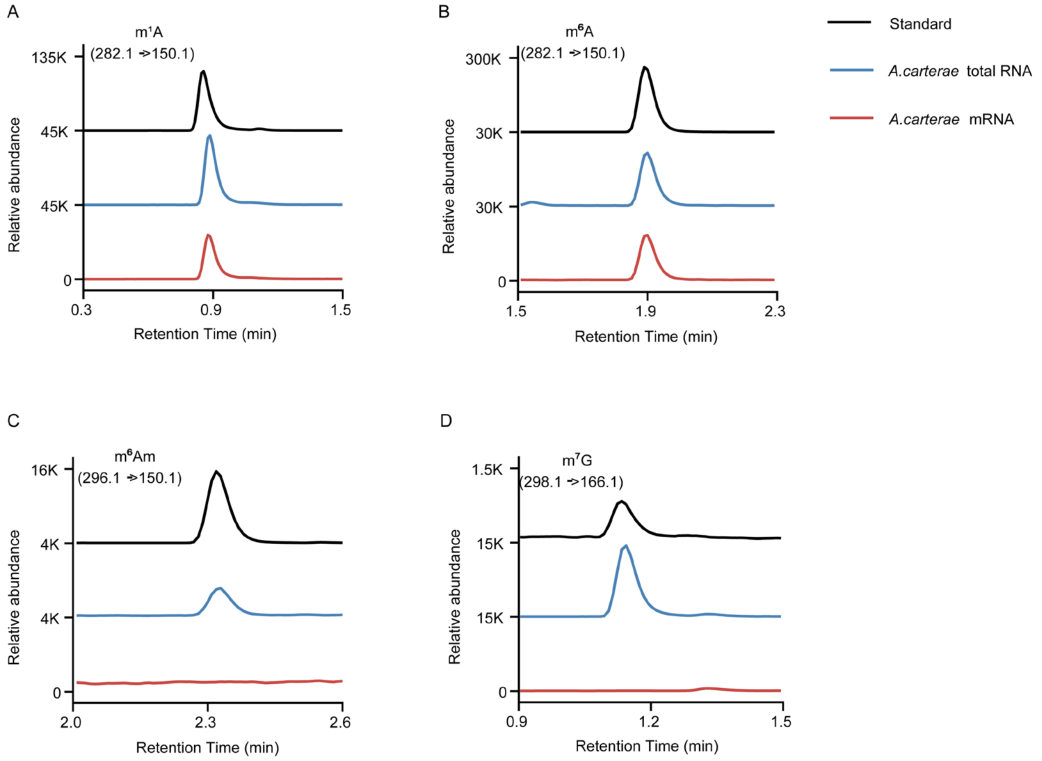

Appendix Figure S1. Common RNA modification species detected in isolated total RNA and mRNA of dinoflagellate *A. carterae*. (A-D) RNA spectra of m^1A, m^6A, m^6A_m and m^7G present in standard samples, *A. carterae* total RNA and mRNA respectively.

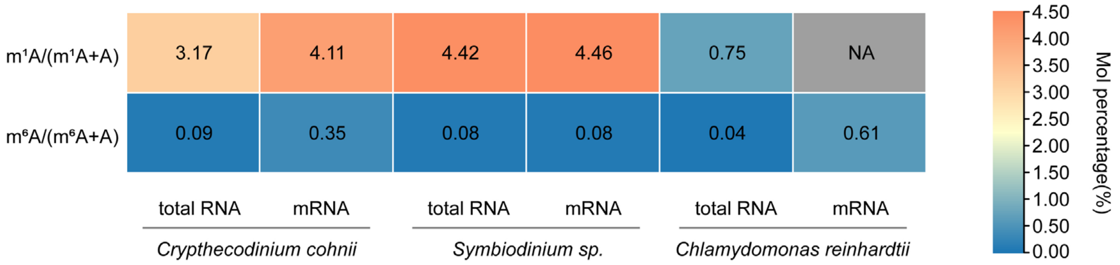

Appendix Figure S2. Quantitative analysis of m^1A and m^6A level in total RNA and mRNA in other dinoflagellates and green algae *Chlamydomonas reinhardtii*. NA, abbreviation of 'not available', means the m^1A level in mRNA of *Chlamydomonas reinhardtii* is below the detection limitation of standards. Sample size = 2 biological replicates for each group.

A

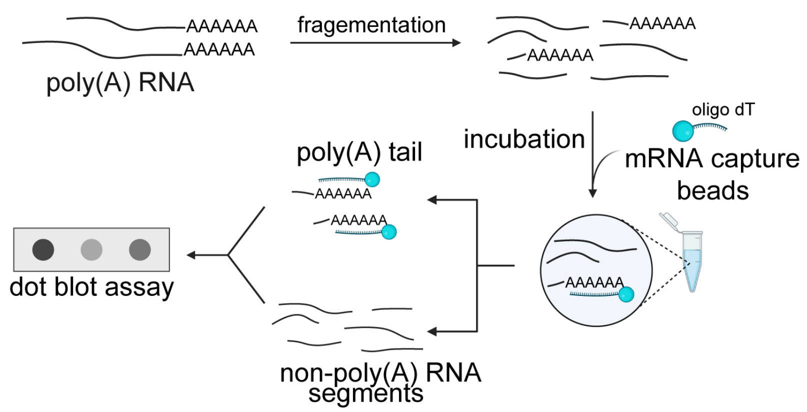

B

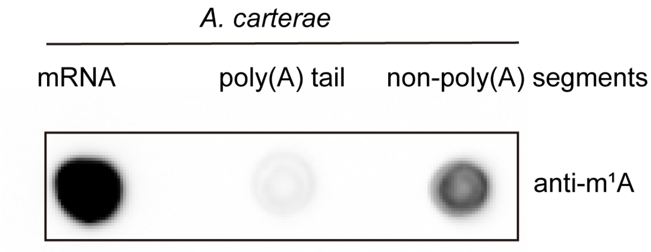

Appendix Figure S3. Distribution of m^{1}A in mRNA poly(A) tails and non-poly(A)-segments. (A) Scheme for isolation of poly(A) tail and non-poly(A) segments from *A. carterae* mRNA. (B) Dot-blot assay of the isolated poly(A) tail and non-poly(A) segments from *A. carterae* mRNA using an anti-m^{1}A antibody.

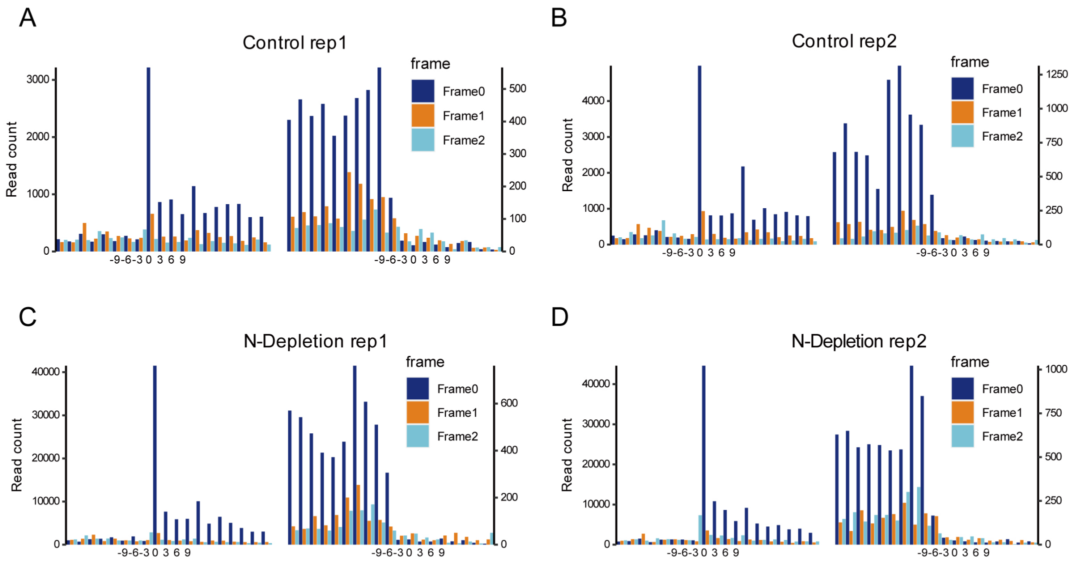

Appendix Figure S4. Quality check for Ribosome profiling data. Ribosome's P-site signals of RPFs for different coding frames near the translation start sites and translation end sites were analyzed. The results displayed a strong 3-nucleotide (nt) periodicity, which is an indicator of high-quality Ribo-seq data. Rep1 and Rep2 represents biological replicates for *A. carterae* Ribo-seq samples under control (A-B) and N-depletion (C-D) conditions respectively.
