# Supplementary material for: Abundant mRNA m1A modification in dinoflagellates: a new layer of gene regulation
Source: EMBO Rep. 2024 Sep 2;25(11):5. doi: 10.1038/s44319-024-00234-2 (PMC11549093; doi:10.1038/s44319-024-00234-2)
Supplement: Supplementary file 8 — Expanded View Figures [file 44319_2024_234_MOESM8_ESM.pdf]

Expanded View Figures

Figure EV1. Characterization of the distribution of m^{1}A in the dinoflagellate mRNA.

(**A**) Counts of MeRIP-identified m^{1}A peaks in different regions of the mRNA. When a peak overlaps with either the start codon or stop codon, we assigned these peaks to the "start codon" and "stop codon" regions, respectively. Otherwise, these peaks were considered to localize in the 5′UTR, CDS and 3′UTR according to their positions. (**B**) Comparison of misincorporation rates for all identified m^{1}A sites in IP samples and input using the m^{1}A-TGIRT-seq approach. (**C**) Snapshot of m^{1}A-mediated misincorporation detected by m^{1}A-TGIRT-seq within the 28s rRNA in *A carterae*. This m^{1}A site is known as m^{1}A$_{1322}$ in the 28s rRNA of mammalian and yeast cells. The y-axis represents the sequencing coverage depth. (**D**) The Venn diagram showing the number of overlapping genes identified by both m^{1}A-seq-TGIRT and MeRIP under normal growth conditions. (**E**, **F**) GO and KEGG pathway enrichment analysis of m^{1}A-methylated genes identified by m^{1}A-TGIRT-seq. *p*-values were obtained using the hypergeometric test. Source data are available online for this figure.

 © The Author(s)

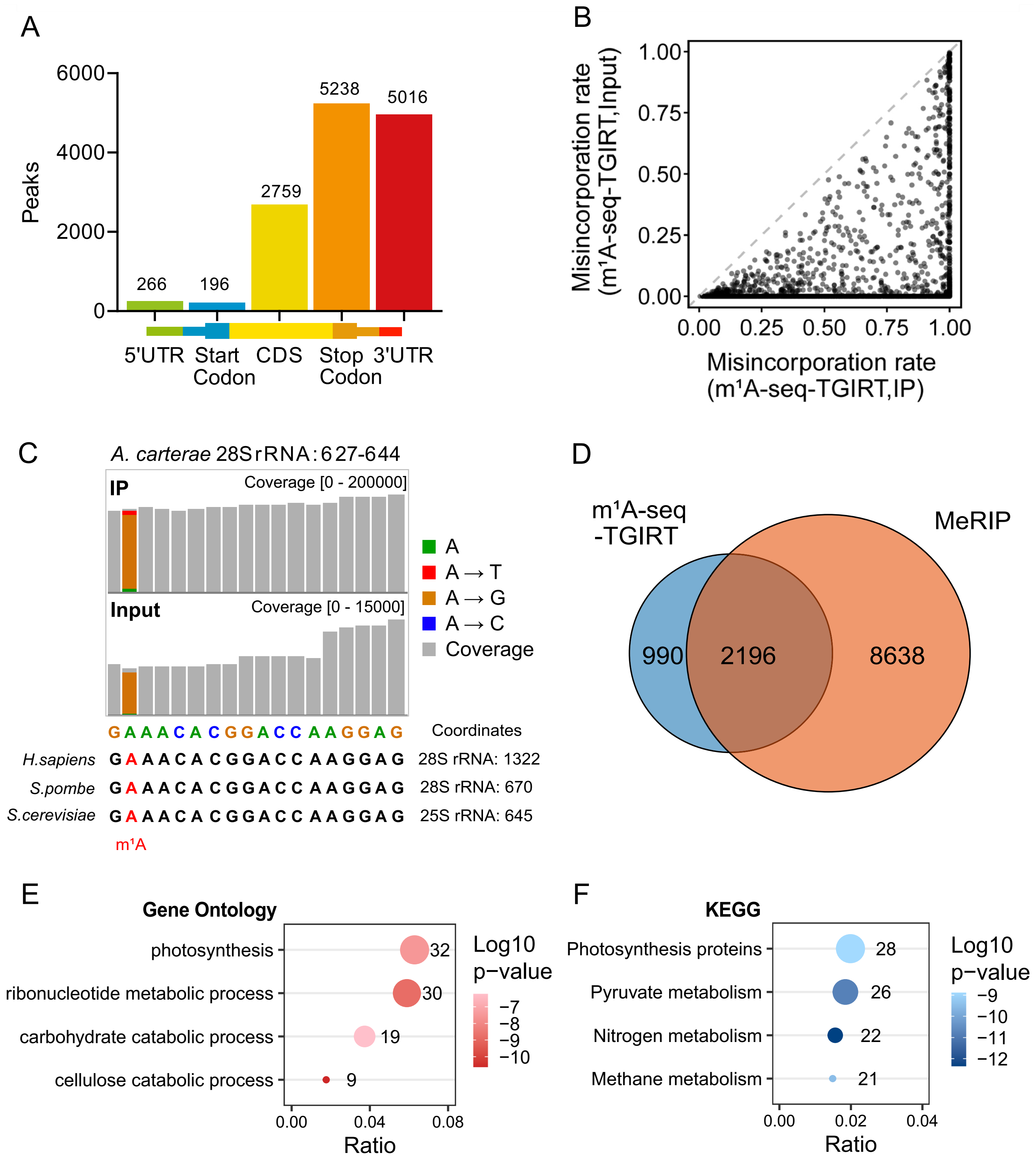
A
Peaks
6000
4000
2000
0
266
196
2759
5238
5016
5'UTR Start Codon CDS Stop Codon 3'UTR
B
Misincorporation rate (m¹A-seq-TGIRT,Input)
1.00
0.75
0.50
0.25
0.00
Misincorporation rate (m¹A-seq-TGIRT,IP)
C
A. carterae 28S rRNA: 627-644
IP Coverage [0 - 200000]
Input Coverage [0 - 15000]
A
A → T
A → G
A → C
Coverage
G A A A C A C G G A C C A A G G A G Coordinates
H.sapiens G A A A C A C G G A C C A A G G A G 28S rRNA: 1322
S.pombe G A A A C A C G G A C C A A G G A G 28S rRNA: 670
S.cerevisiae G A A A C A C G G A C C A A G G A G 25S rRNA: 645
m¹A
D
m¹A-seq -TGIRT
MeRIP
990
2196
8638
E
Gene Ontology
photosynthesis
ribonucleotide metabolic process
carbohydrate catabolic process
cellulose catabolic process
32
30
19
9
Log10 p−value
−7
−8
−9
−10
0.00 0.04 0.08
Ratio
F
KEGG
Photosynthesis proteins
Pyruvate metabolism
Nitrogen metabolism
Methane metabolism
28
26
22
21
Log10 p−value
−9
−10
−11
−12
0.00 0.02 0.04
Ratio

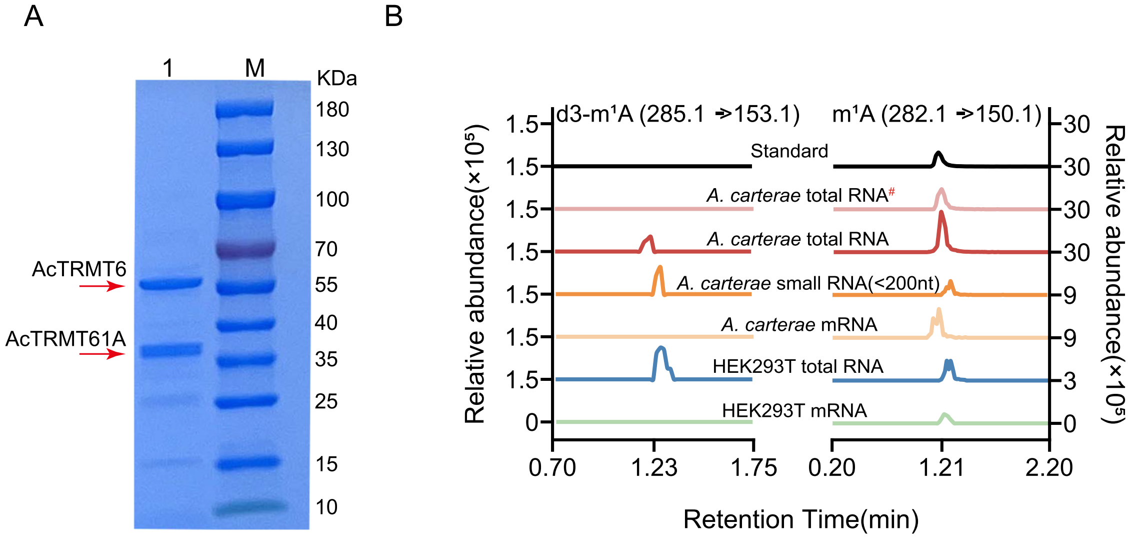

Figure EV2. The methyltransferase activities of AcTRMT6/AcTRMT61A heterocomplex to different RNA substrates.

(**A**) SDS-PAGE analysis showing the purified AcTRMT6/AcTRMT61A complex with expected molecular weights of 38.5 kDa (AcTRMT61A) and 51.5 kDa (AcTRMT6), respectively. Lane 1, the purified recombinant AcTRMT6/AcTRMT61A heterocomplex; Lane M, the Thermo Scientific PageRuler Plus Prestained Protein Ladder. (**B**) LC-MS/MS spectra of various RNA substrates from in vitro methylation reaction. #, represents the addition of heat-inactivated AcTRMT6/AcTRMT61A heterocomplex in this reaction mixture. The native recombinant protein complex AcTRMT6/AcTRMT61A were used in all other reactions.

 © The Author(s)

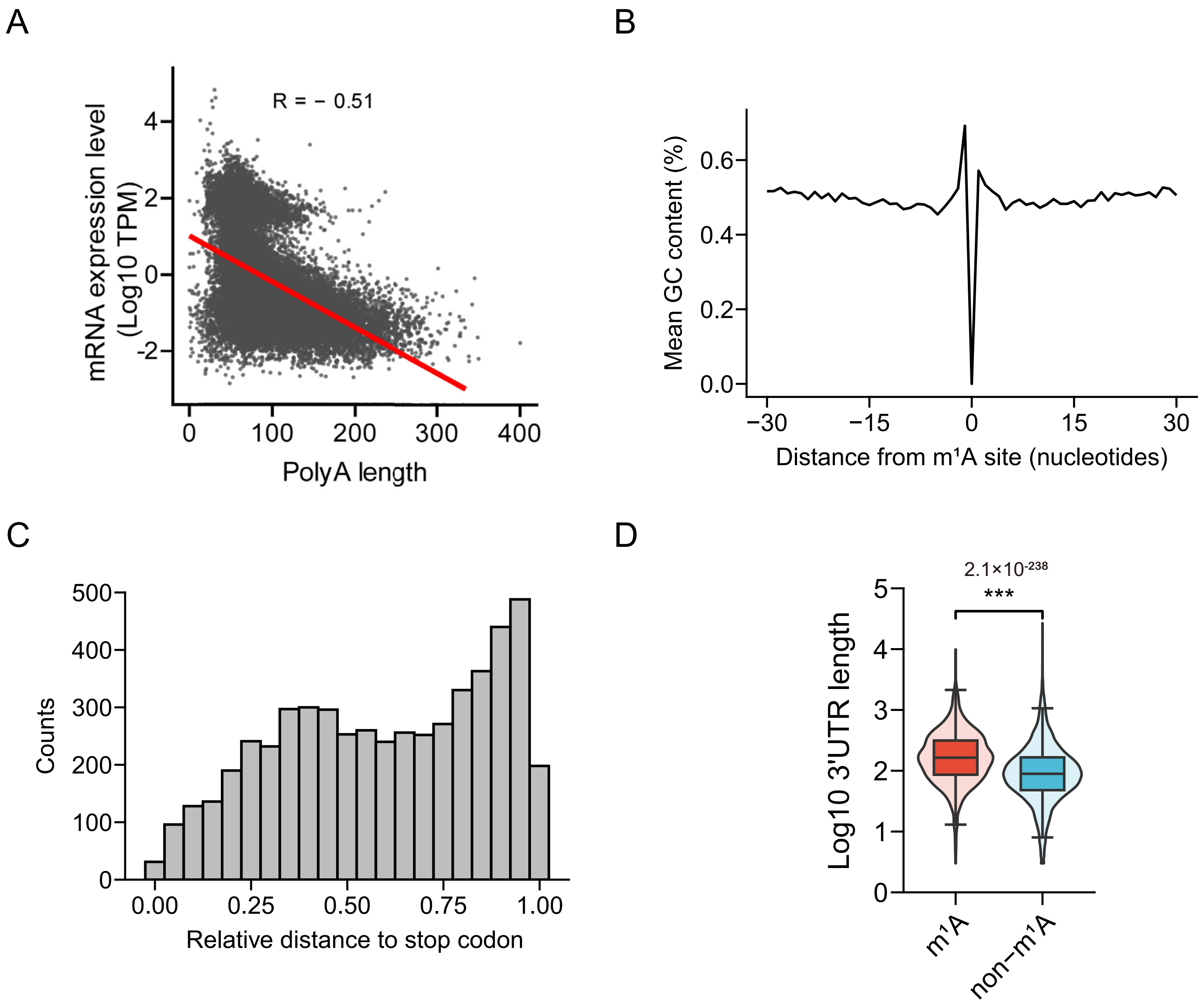

Figure EV3. The correlation between gene expression level and poly(A) tails, and detailed characteristics of m¹A-methylated transcripts.

(**A**) Correlation analysis of mRNA abundance with the poly(A) length of encoded transcripts in *A. carterae* (Pearson correlation test, $p < 2.2 \times 10^{-16}$). The distribution properties of m¹A sites within the 3′UTR (same sites used in Main Fig. 3) are shown in following Figures (**B–D**). (**B**) The mean GC content of neighboring nucleotides around all identified m¹A sites ($n = 5297$) within the 3′UTR. (**C**) The histogram showing the distribution pattern of the relative distance to the stop codon for all identified m¹A sites within the 3′UTR. (**D**) A comparison of 3′UTRs' length of genes with m¹A-methylated 3′UTRs and those with unmethylated ones (t-test (two-sided), ***$p < 0.001$). The median value in each group is indicated by a center line, with the box representing the upper and lower quartiles, and whiskers indicating the 1.5× interquartile range. Note: For EV3B–D, the m¹A-TGIRT-seq experiment was carried out with two independent biological replicates ($n = 2$). Source data are available online for this figure.

© The Author(s)

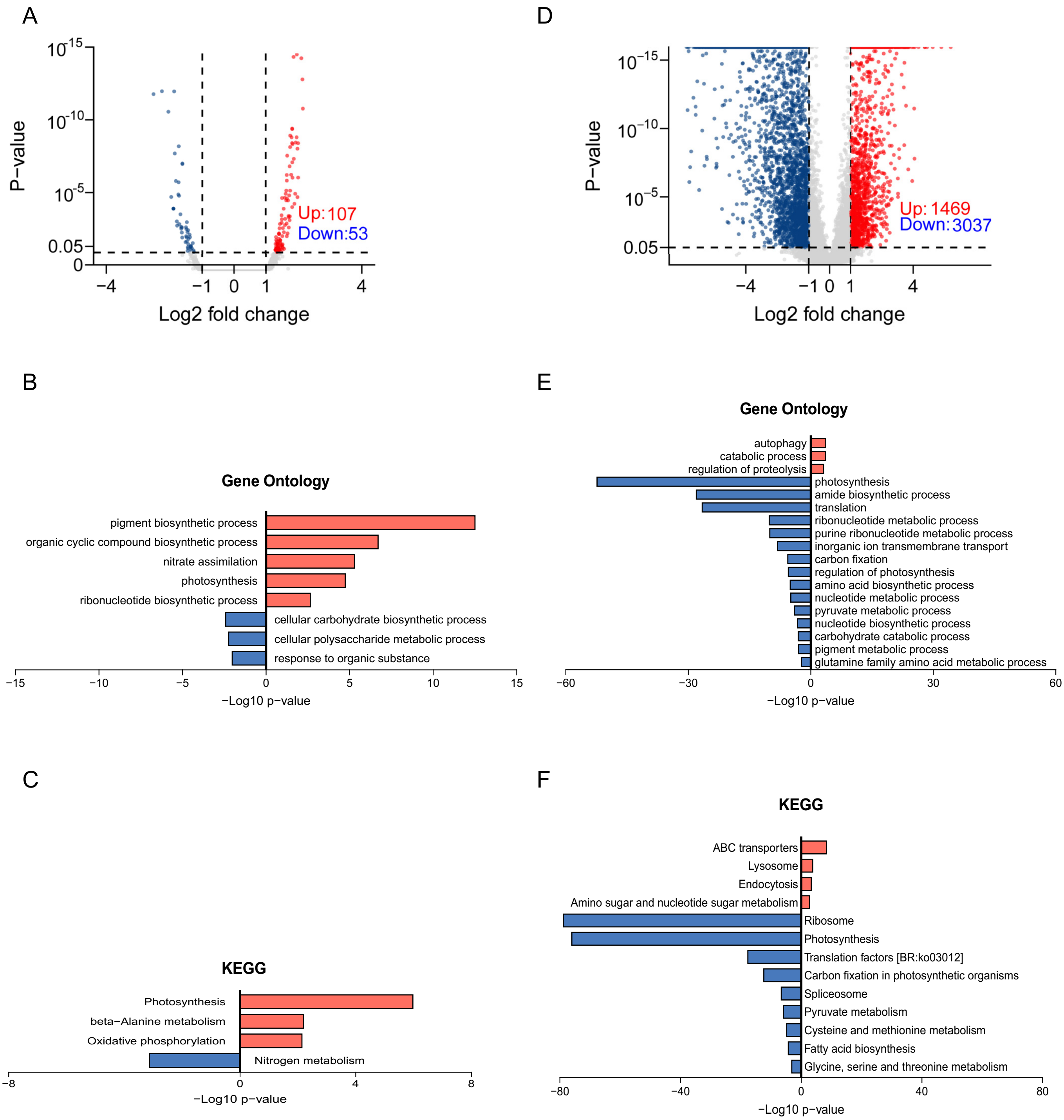

Figure EV4. N-depletion in the dinoflagellate *A. carterae* induces minor differences in mRNA accumulation levels but dramatic changes in translation efficiency.

(**A**) Volcano plot showing differentially expressed genes (DEGs) under N-depletion treatment. (**B**, **C**), GO and KEGG enrichment analysis revealing the enriched GO terms and pathways of these DEGs, respectively. (**D**) Volcano plot showing differential translation efficiency genes (DTEGs) after N-depletion treatment. (**E**, **F**) Functional analysis of these DTEGs, respectively. Red colors indicate either upregulated DEGs or DTEGs, while blue colors represent either downregulated DEGs or DTEGs. *p*-values were calculated using the Wald test (**A**), Hypergeometric test (**B**, **C**, **E**, **F**) and Chi-Squared Test (**D**).

 © The Author(s)

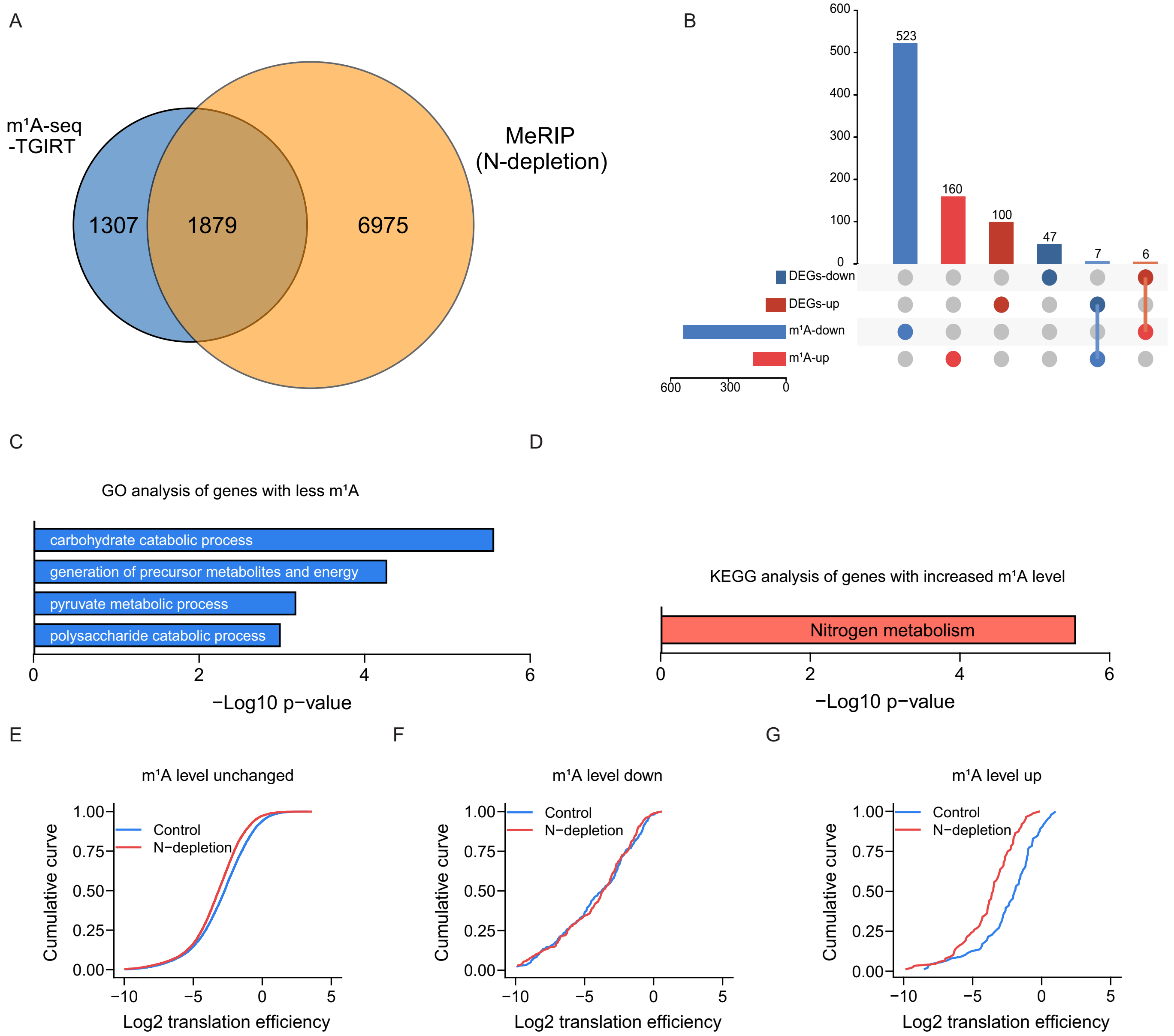

Figure EV5. Features of differentially methylated m¹A genes under N-depletion.

(**A**) The Venn diagram depicting the overlap of genes identified as methylated by both m¹A-seq-TGIRT and MeRIP under N-depletion conditions. (**B**) The Upset plot showing the numbers of shared genes between DEGs and differentially methylated genes. (**C**, **D**) GO and KEGG enrichment analysis of the downregulated and upregulated m¹A-modified genes, respectively. *p*-values were calculated using hypergeometric test. (**E**–**G**) The translation efficiency is plotted as accumulative fractions for genes with unchanged methylation (**E**), decreased methylation (**F**), and elevated methylation (**G**) for both control and N-depletion treatment groups.

© The Author(s)
